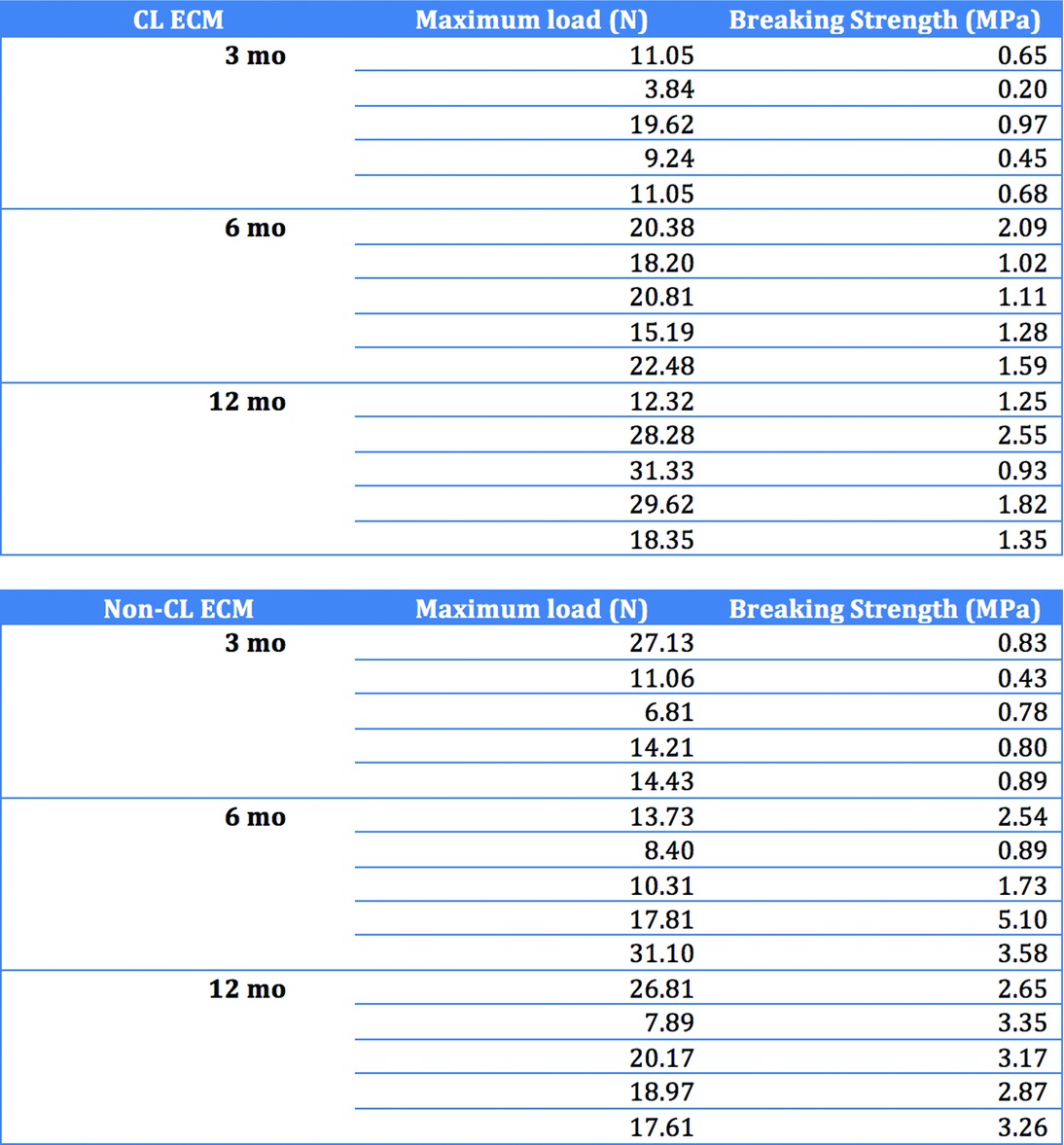# Comparison of Cross-linked and Non–Cross-linked Acellular Porcine Dermal Scaffolds for Long-term Full-Thickness Hernia Repair in a Small Animal Model

**Published:** 2014-06-17

**Authors:** Ondrej Mestak, Zuzana Spurkova, Kamila Benkova, Pavel Vesely, Veronika Hromadkova, Jakub Miletin, Robert Juzek, Jan Mestak, Martin Molitor, Andrej Sukop

**Affiliations:** ^a^Department of Plastic Surgery, 1st Medical Faculty of Charles University in Prague, Bulovka Hospital; ^b^Department of Pathology, Bulovka Hospital; ^c^Department of Plastic Surgery, 3rd Medical Faculty of Charles University in Prague, University Hospital Kralovske Vinohrady, Srobarova, Prague, Czech Republic

**Keywords:** extracellular matrix, biologics, biocompatibility, cross-linking, hernia

## Abstract

**Background:** This study compared the strength of incorporation and biocompatibility of 2 porcine-derived grafts (cross-linked and non–cross-linked) in a rat hernia model. **Methods:** A standardized 2 × 4 cm^2^ fascial defect was created in 30 Wistar rats and repaired with either a cross-linked or a non–cross-linked graft. The rats were killed 3, 6, and 12 months later. The strength of incorporation, vascularization, cellular invasion, foreign body reaction, and capsule formation were evaluated. **Results:** Both graft materials showed cellular ingrowth and neovascularization by 3 months postimplantation. The average level of cellularization was significantly higher in the non–cross-linked grafts than in the cross-linked grafts at 6 months (2 vs 1; *P* = .029). Vascularization was significantly higher in the non–cross-linked grafts than in the cross-linked grafts at 6 months postimplantation (2 vs 1; *P* = .029) and insignificant at 3 months (2 vs 1.75; *P* = .311) and 12 months (1 vs 0.67; *P* = 1). The maximum load and breaking strength of both biomaterials increased during the study period. Overall, the strength of incorporation of the non–cross-linked grafts increased from 3 months (0.75 MPa) to 12 months (3.06 MPa) postimplantation. The strength of incorporation of the cross-linked grafts also increased from 3 months (0.59 MPa) to 12 months (1.58 MPa) postimplantation. **Conclusions:** The results of our study suggest that non–cross-linked grafts may be slightly more biocompatible and allow a more rapid and higher degree of cellular penetration and vascularization, resulting in stronger attachment to the tissues.

Implantable extracellular matrix (ECM) derived from animal tissues are used in modern surgery, including those derived from human dermis (AlloDerm, LifeCell, NJ), porcine dermis (Permacol, Covidien, Ireland), porcine small intestinal submucosa (Surgisis, Cook Surgical), or from bovine pericardium (Veritas, Synovis Surgical Innovations, MN). These biomaterials consist primarily of collagen structures and have a minor component of glycoproteins, which helps maintain the structure of the original intercellular tissue. In addition, they are vascularized and well integrated into the host tissue. This process enables their use even in infected areas, unlike synthetic materials, which have a high risk of acquiring infection,^1^ skin erosion, fistula formation, or bowel obstruction.^2^^-^4 If a synthetic mesh is used in a contaminated field, 50% to 90% of the patients require its eventual removal .^5^^,^^6^ Extracellular matrices are frequently used for hernia treatment,^7^^-^10 often in conjunction with component separation,^11^ and in breast reconstruction, to increase the coverage of an implant.^12^

According to their structures, these materials are categorized as CL or non-CL. The main reason to cross-link materials is to increase their durability. The disadvantage of cross-linking is that it decreases biocompatibility and the strength of incorporation (SOI) into the host's tissues. There is an ongoing discussion about how much these properties are affected by cross-linking.^10^^,^^13^^-^16

The aim of our study was to assess biocompatibility and the SOI of CL ECM and non-CL ECM grafts in a long-term animal model of ventral hernia.

## MATERIALS AND METHODS

This study was conducted in strict accordance with the recommendations in the Guide for the Care and Use of Laboratory Animals of the Ministry of Agriculture of the Czech Republic. The protocol was approved by the Committee on the Ethics of Animal Experiments of the first Medical Faculty of Charles University in Prague. All of the surgery was performed under anesthesia, and all efforts were made to minimize suffering.

### Materials used

We used non-CL ECM material (Medicem Technology, Czech Republic) that was produced from split-thickness porcine skin grafts. The material was treated with trypsin (0.3%) to remove the cells, dehydrated by desiccation while attached to a glass substrate, and sterilized by gamma irradiation. We compared this non-CL ECM material to commercially available CL ECM Permacol (Covidien, Ireland). Permacol is one of the most widely used and well-studied surgical dermal scaffolding material.^9^^,^^17^^,^^18^ It is a porcine-derived acellular dermal scaffold manufactured by trypsinization (to remove all of the living cells), solvent-extraction (to remove all of the lipids and fat deposits), gamma irradiation and cross-linking using hexamethylene diisocyanate.

### Surgical procedure

Female Wistar rats (n = 30) weighing 340 to 400 g were anesthetized with an intramuscular injection of 50 mg/kg of body weight of Zoletil (Virbac Laboratories, France) 30 minutes prior to surgery. After shaving the abdominal area and applying an antibiotic solution, a midline incision (5 cm^2^) through the skin and subcutaneous tissue of the abdomen was made. Then, we performed carinal-shaped full-thickness excision (4 × 2 cm^2^) of the abdominal wall. The rats were randomly divided into 2 groups. The first group (n = 15) received CL-ECM grafts to repair their abdominal walls. The second group (n = 15) received non-CL ECM grafts to repair their abdominal walls. We cut carinal-shaped pieces of each material that measured 4 × 2 cm^2^. These materials were sutured to the edges of the abdominal wall defects to patch them, using continuous sutures of Prolene 4/0 (Ethicon Inc, USA). The wounds were closed with interrupted sutures of Vicryl 3/0 (Ethicon Inc, USA). Postoperative analgesia was assured by an intramuscular injection of 0.01 mg of Butomidor (Richter Pharma, Austria) per rat. Because of death of 1 animal from the CL ECM group shortly after the operation and a wound dehiscence in 1 animal from each group, we consequently implanted 2 more animals for the CL ECM group and 1 animal for the non-CL ECM group. The postoperative course and observation period was without other undesirable events.

**Figure 1:**
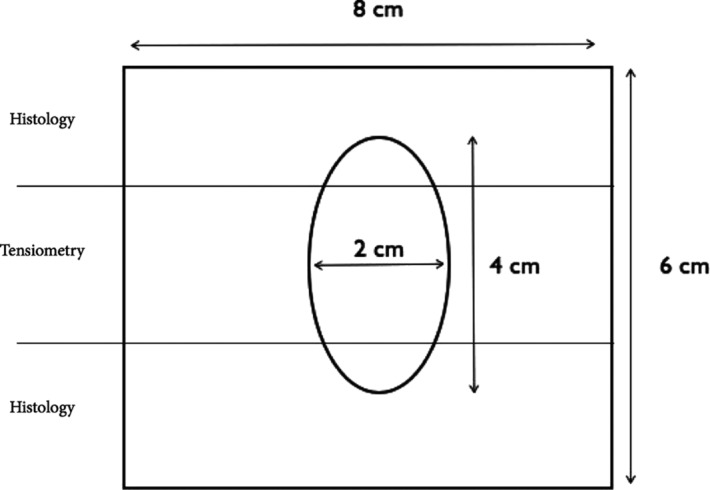
A square block of the abdominal wall measuring 8 × 6 cm^2^ was excised en bloc and divided into 3 parts. The cranial and caudal portions were processed for histological evaluation. The middle portion of the excision (a 15-mm-wide strip) was transported in saline solution for tensile tests.

Five animals from each group were killed 3, 6, and 12 months postimplantation by an intramuscular injection of Ketamine (0.1 mL/100 g) and Medetomidine (0.01/100 g) and an intracardial injection of T61 (3 mL/100 g). The incidence of herniation was determined. A square block of abdominal wall measuring 8 × 6 cm^2^ was excised en bloc and divided into 3 portions (Fig 1). The cranial and caudal portions were processed for histological evaluation. The middle portion of the excision (a 15-mm-wide strip) was transported in saline solution for tensile tests.

### Histological examination

**Figure 2:**
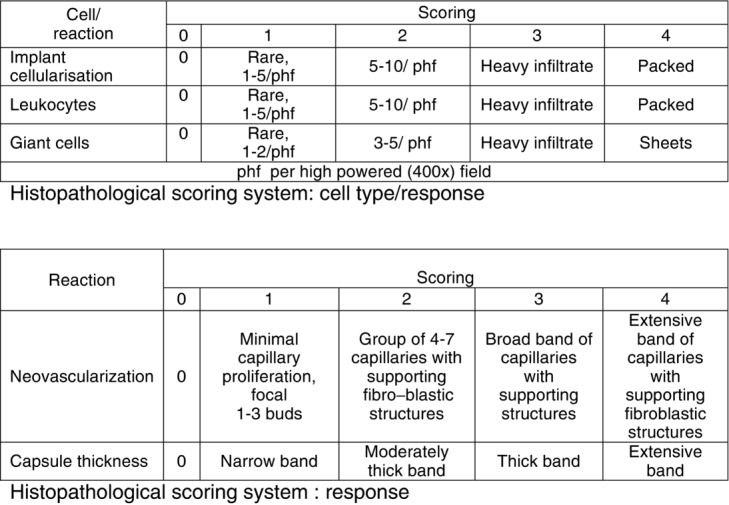
Semiquantitative histopathological evaluation of each explant, as described in ISO 10993-6.

After explantation, the tissue samples were fixed in formaldehyde, stained with hematoxylin and eosin and subjected to histological examination using an Olympus BX50 microscope with 40x, 100x, and 200x magnification. Semiquantitative histopathological evaluation of each explant was performed using the scoring system described in ISO 10993-6 (Fig 2). The scores for each parameter of each animal in a group were added to obtain a sum, which was divided by the number of animals in the group to obtain a test or reference group average. The samples were scored independently by 2 pathologists (K.M. and Z.S.).

### Mechanical properties

The SOI was evaluated using an Inspekt Table 10 kN with a 100 N load cell and Labmaster software (Hegewald and Peschke, Germany). The preload was set to 0.05 N, and the speed of crosspiece movement was set to 200 mm/min. The thickness of the materials was evaluated using a Mitutoyo Absolute digital caliper (Mitutoyo Inc, Japan). Each specimen was oriented horizontally with the ends secured by grips. We evaluated SOI and the site of the specimen's rupture (implant, implant-tissue conjunction, tissue). The degree of SOI was expressed as the breaking strength recorded in megapascal. The breaking strength was the maximum tension developed across the fascia-patch interface related to cross-section of the specimen before it ruptured. We also evaluated the maximum load recorded in Newtons.

The data were analyzed statistically; quantitative data using Wilcoxon test, and categorical data using nonparametric Pearson Chi-Square, Mann-Whitney test and Fisher exact test.

## RESULTS

### Local findings upon explantation

There were no seromas near the engrafted materials. No hernias were observed at the time of explantation in either group. Some of the animals in the non-CL ECM group had slight abdominal bulging (1/5 at 3 months, 2/5 at 6 months, 1/5 at 12 months). There were no bowel adhesions in either group. We observed only omental adhesions on the peritoneal side of the engrafted materials. The incidence of these adhesions was evenly distributed between both groups. Starting 3 months postimplantation, all of the graft materials had macroscopic evidence of early vascularization, with blood vessels on both sides. There were no signs of inflammation, fibrosis, calcification, or granulomatous reaction. The graft materials were stably integrated with the subcutaneous tissues and the abdominal wall. The non-CL ECM materials displayed significant degradation at the macroscopic level, with the appearance of thin, almost transparent membranes at 3 months postimplantation. The degradation had not progressed by 6 and 12 months postimplantation. The CL ECM materials did not exhibit any macroscopically obvious degradation throughout the study period. At the explantation, CL ECM materials appeared as they did when they were implanted.

### Histology

**Figure 3:**
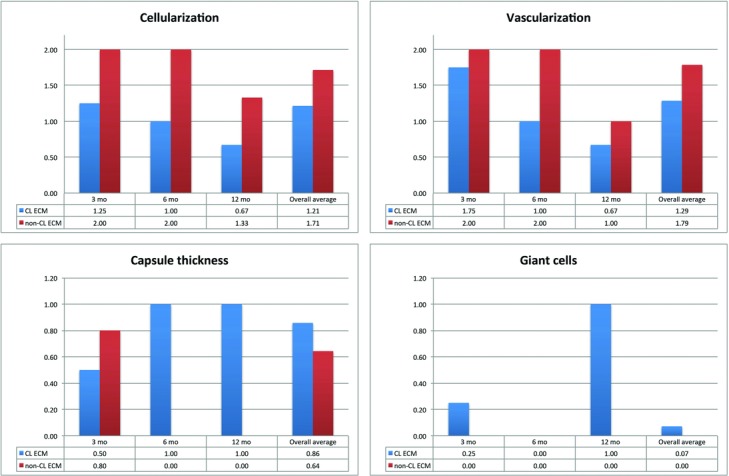
Graphs showing the average cellularization, vascular density, capsule thickness, and number of giant cells in both types of graft materials. The average level of cellularization was significantly higher in the non-CL ECM grafts than in the CL ECM grafts at 6 months (2 vs 1; *P* = .029). The difference in cellularization was insignificant at 3 months (2 vs 1.25; *P* = 0.549) and 12 months (1.33 vs 0.67; *P* = .329). Vascularization was significantly higher in the non-CL ECM grafts than in the CL ECM grafts at 6 months postimplantation (2 vs 1; *P* = .029) and insignificant at 3 months (2 vs 1.75; *P* = .311) and 12 months (1 vs 0.67; *P* = 1). The capsules in the non-CL ECM were significantly thinner in the non-CL ECM at 6 months (0 vs 1; *P* = .005) and 12 months (0 vs 1; p = 0.009), compared to the capsules surrounding the CL-ECM grafts. Giant cells were missing in non-CL ECM and were present in CL ECM at 3 months and 12 months, but this result was not statistically significant. CL indicates cross-linked; ECM, extracellular matrix.

**Figure 4:**
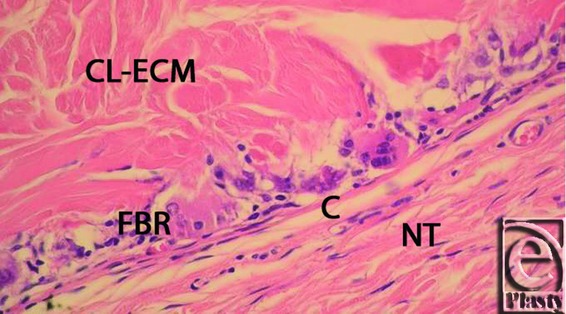
CL-ECM graft 3 months after implantation in the animal model. A fine capsule (C) exists at the interface of the normal tissue (NT) and the implant. Foreign body reaction (FBR) is apparent. The implant has no signs of inflammation, capillary formation and or fibroblast penetration. CL indicates cross-linked; ECM, extracellular matrix.

**Figure 5:**
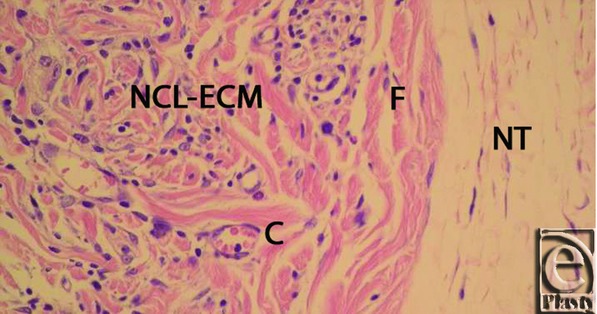
Non–CL-ECM graft 3 months after implantation in the animal model. Relatively strong vascularization of the implant has occurred. Fibroblasts have penetrated the implant. Capillaries (C) are located in foci. No distinct fibrous capsule, only sporadic incoherent fibrosis, has formed. Normal tissue (NT). CL indicates cross-linked; ECM, extracellular matrix.

Both ECM materials showed cellular ingrowth and neovascularization by 3 months postimplantation (Table 1). The average level of cellularization was significantly higher in the non-CL ECM than in the CL ECM at 6 months (2 vs 1; *P* = .029). The difference in cellularization was insignificant at 3 months (2 vs 1.25; *P* = .549) and 12 months (1.33 vs 0.67; *P* = .329) (Fig 3). Vascularization was significantly higher in the non-CL ECM than in the CL ECM at 6 months postimplantation (2 vs 1; *P* = .029) and insignificant at 3 months (2 vs 1.75; *P* = 0.311) and 12 months (1 vs. 0.67; *P* = 1). The blood vessels and cells in the CL ECM were located almost entirely at the implant-musculofascial interface, whereas the cells and blood vessels in the non-CL ECM were located more centrally. The matrix structure was intact in both groups at all of the time points. The capsules surrounding the non-CL ECM were thicker at 3 months (0.8 vs 0.33), but this result was insignificant (*P* = 0.486). The capsules in the non-CL ECM were significantly thinner in the non-CL ECM at 6 months (0 vs 1; *P* = 0.005) and 12 months (0 vs. 1; *P* = 0.009), compared to the capsules surrounding the CL-ECM, suggesting that capsule-thickening might be reversible (Figs 4, and 5).

### Mechanical properties

**Figure 6:**
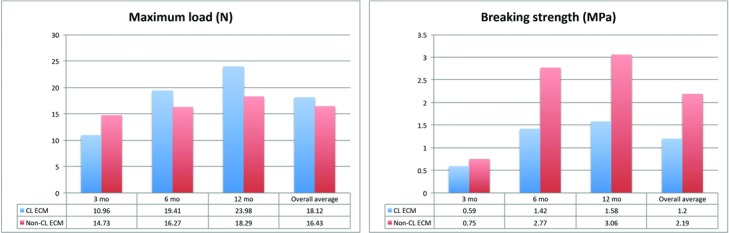
Graphs showing the increasing maximum load and breaking strength that developed in both materials during the study period.

**Figure 7:**
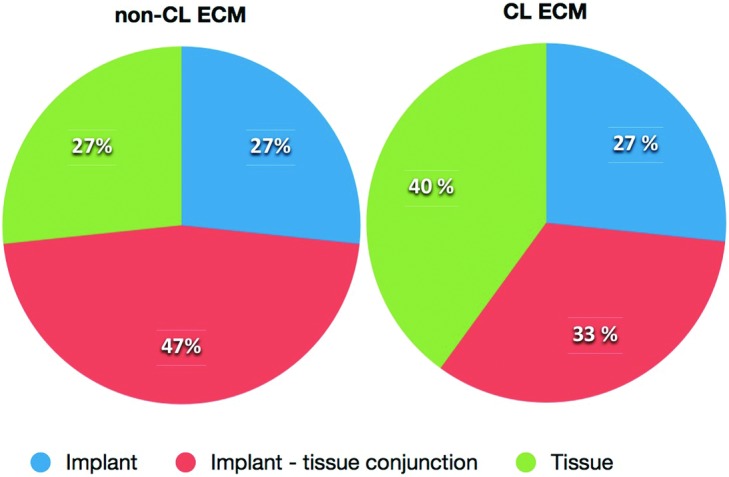
Graphs showing the site of rupture. It was evenly distributed among the implant, the implant-tissue conjunction, and the tissue.

The maximum load and breaking strength of both biomaterials increased during the study period (Fig 6 and Table 2). The sites of rupture were evenly distributed among the implant, the implant-tissue conjunction region, and the tissue (Fig 7). At 3 months postimplantation, non-CL ECM had significantly larger SOI (0.75 vs 0.59 MPa; *P* = .007). At 6 months postimplantation, the differences of SOI between the non-CL ECM and the CL ECM were insignificant (2.77 vs 1.42 MPa; *P* = .386) and stayed insignificant at 12 months postimplantation (3.06 vs 1.58 MPa; *P* = .083). Overall, the SOI of the non-CL ECM increased from 3 months (0.75 MPa) to 12 months (3.06 MPa) postimplantation. The SOI of the CL ECM also increased from 3 months (0.59 MPa) to 12 months (1.58 MPa) postimplantation.

## DISCUSSION

Extracellular matrices are materials that possess excellent biocompatibility and an ability to incorporate into the host's tissues. They undergo vascularization, which increases their resistance to infection. Depending on their chemical and physical processing, these biomaterials can be reasonably biocompatible and sufficiently stable for long-term tissue replacement (eg, abdominal wall reconstruction, thoracic wall reconstruction, breast reconstruction). Their biostability can be increased by cross-linking. However, the attempts of ECMs producers to improve the firmness of meshes by cross-linking them reduced their biocompatibility and the strength of their attachment to host tissues. The materials are too compact for vessels and other host tissues to grow in them, resulting in a weak connection between the material and host tissues such as the abdominal wall and in a low resistance to infection.

Many reagents, from aldehyde to hexamethylene diisocyanate, have been used for cross-linking.^19^^,^^20^ Most modern biomaterials currently used in clinical practice are not covalently CL. Reviewing a list of ECM materials used in clinical practice shows that most of them lack this type of processing.^21^

The aim of our study was to assess biocompatibility and the SOI of CL ECM and non-CL ECM grafts in a long-term animal model of ventral hernia.

The results of our study suggested that non-CL ECMs were slightly more biocompatible and allowed a more rapid and higher degree of cellular penetration and vascularization over CL ECMs. Higher cellularization and vascularization leads to higher resistance against infection, which is clinically most important benefit of acellular dermal matrices. In addition, results of tensiometry indicated that non-CL ECMs had stronger attachments to tissues (SOI) over CL ECMs.

Our study stands somewhere in between present experimental studies comparing the use of non-CL versus CL ECM. Some studies, for example, de Castro et al,^13^ Macleod et al,^17^ Smart et al,^21^ Gaertner et al,^22^ and O’Brien et al,^23^ suggest that CL ECM last longer in vivo and have excellent biocompatibility. However, authors such as Butler et al,^10^ Ayubi et al,^18^ and Wotton^24^ suggest that CL ECM are less biocompatible and not as applicable in clinical practice and, furthermore, may actually be at risk of rejection.^25^ The companies that sell these products funded significant parts of these studies. In addition, most of these studies had been testing materials for a period of several weeks or months^14^^,^^16^^,^^18^^,^^22^ contrary to our study, which has been conducted for a period of 12 months.

Extracellular matrices have been widely studied on animal models. Most of available data come straight from producers and had not been peer reviewed. Different animals were used as animal models, such as pig,^14^ primate, or guinea pig.^10^ By fat the most common animal for use as animal model of incisional hernia has been rat.^13^^,^^18^^,^^22^ Specimens from these small animal models are sufficient for histological examination and mechanical testing.

We found that the non-CL ECM implants were macroscopically degraded by 3 months postimplantation, but the degradation did not progress between 3 and 12 months postimplantation. We expected a correlate of the macroscopical thinning of the grafts in their SOI outcomes. However, mechanical testing showed both the non-CL ECM and CL ECM had the same mechanical properties. These results demonstrate that the 3-dimensional structure, collagen fiber orientation, and composition of the implant material are more important than its thickness. The structures responsible for the mechanical firmness of the non-CL ECM material are most likely more resistant to degradation than are the structures that are less important for mechanical firmness. Hernia repairs performed with mesh generally fail at the mesh-fascia interface rather than within the mesh itself. The early establishment of a strong connection between the mesh and fascia is important to reducing the recurrence of a hernia due to the separation of the mesh-fascia interface.^10^

The maximum load and breaking strength of both biomaterials increased during the study period. This trend is related to their continued integration into the tissue of abdominal wall and to the remodeling of the implant in response to mechanical stress.^26^

None of the animals in our study developed a hernia. Nevertheless, some of the animals in non-CL ECM group developed bulges in the graft material, which was likely induced by the postimplantation degradation of the biomaterial and structural flaws in the biological biomaterials.

## CONCLUSIONS

Both materials maintained their strength throughout the study period. The macroscopic and microscopic evaluations revealed excellent integration with the tissues. The greater extent of tissue ingrowth observed upon histologic examination was related to the greater SOI. Our study suggested that these materials are suitable for abdominal wall reconstruction. Non-CL ECM grafts are more biocompatible and allow a more rapid and higher degree of cellular penetration and vascularization, resulting in stronger attachment to the tissues.

## Figures and Tables

**Table 1:** Results of individual measurements of histological parameters at given time points

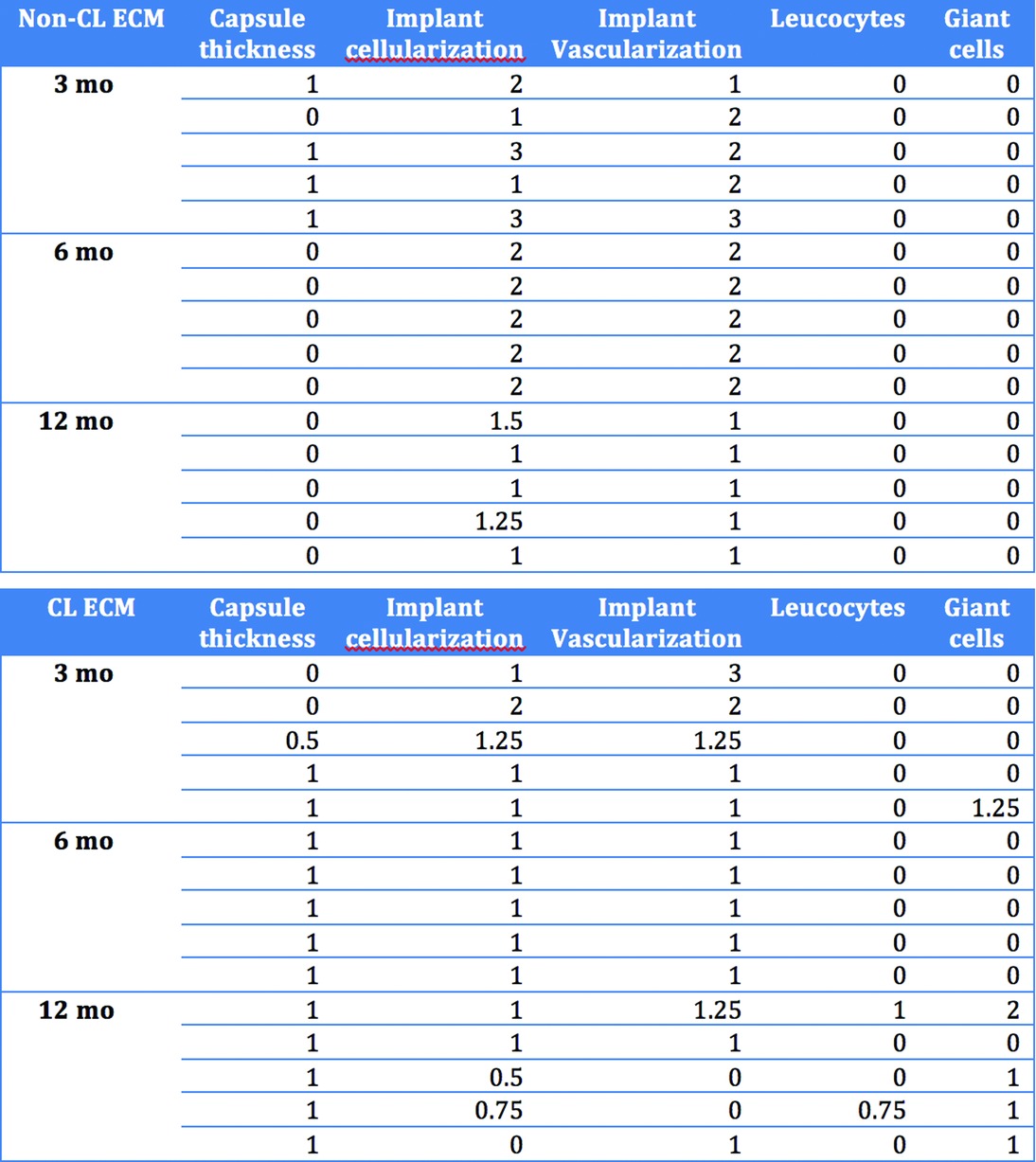

**Table 2:** Results of individual measurements of mechanical parameters at given time points